# Eukaryotic signaling pathways targeted by *Salmonella *effector protein AvrA in intestinal infection *in vivo*

**DOI:** 10.1186/1471-2180-10-326

**Published:** 2010-12-23

**Authors:** Xingyin Liu, Rong Lu, Yinglin Xia, Shaoping Wu, Jun Sun

**Affiliations:** 1 Department of Medicine, Gastroenterology & Hepatology Division, University of Rochester, 601 Elmwood Avenue, Rochester, NY 14642, USA; 2 Department of Biostatistics and Computational Biology, University of Rochester, 601 Elmwood Avenue, Rochester, NY 14642, USA; 3 Department of Microbiology and Immunology, University of Rochester, 601 Elmwood Avenue, Rochester, NY 14642, USA; 4 Wilmot Cancer Center, University of Rochester, 601 Elmwood Avenue, Rochester, NY 14642, USA

## Abstract

**Background:**

The *Salmonella AvrA *gene is present in 80% of *Salmonella enterica serovar *strains. AvrA protein mimics the activities of some eukaryotic proteins and uses these activities to the pathogen's advantage by debilitating the target cells, such as intestinal epithelial cells. Therefore, it is important to understand how AvrA works in targeting eukaryotic signaling pathways in intestinal infection *in vivo*. In this study, we hypothesized that AvrA interacts with multiple stress pathways in eukaryotic cells to manipulate the host defense system. A whole genome approach combined with bioinformatics assays was used to investigate the *in vivo *genetic responses of the mouse colon to *Salmonella *with or without AvrA protein expression in the early stage (8 hours) and late stage (4 days). Specifically, we examined the gene expression profiles in mouse colon as it responded to pathogenic *Salmonella *stain SL1344 (with AvrA expression) or SB1117 (without AvrA expression).

**Results:**

We identified the eukaryotic targets of AvrA and the cell signaling pathways regulated by AvrA *in vivo*. We found that pathways, such as mTOR, NF-kappaB, platelet-derived growth factors, vascular endothelial growth factor, oxidative phosphorylation, and mitogen-activated protein kinase signaling are specifically regulated by AvrA *in vivo *and are associated with inflammation, anti-apoptosis, and proliferation. At the early stage of *Salmonella *infection, AvrA mainly targeted pathways related to nuclear receptor signaling and oxidative phosphorylation. At the late stage of *Salmonella *infection, AvrA is associated with interferon-gamma responses.

**Conclusion:**

Both early and late phases of the host response exhibit remarkable specificity for the AvrA+ *Salmonella*. Our studies provide new insights into the eukaryotic molecular cascade that combats *Salmonella*-associated intestinal infection *in vivo*.

## Background

The capacity of pathogenic *Salmonella *to infect their hosts is often dependent on the ability of *Salmonella *to inject virulent factors directly into the host cell cytosol through the type-three secretion system (TTSS). These injected bacterial proteins, called effectors, are of special interest in studies of host-pathogen interactions because effectors can manipulate host cell function [1,2]. The effectors often have unique functions suited to a particular pathogen's infection strategy.

AvrA is a *Salmonella *effector that is translocated into host cells [3]. The *AvrA *gene is present in 80% of *Salmonella enterica serovar *Typhimurium strains [4]. Previous studies show that AvrA related family members include *Yersinia *virulence factor, YopJ, and the *Xanthomonas campestris pv.vesicatoria *protein, AvrBsT [5]. Analysis with MEROPS database shows that AvrA belongs to YopJ-like proteins and genes (family C55) in bacterial species (see details in http://merops.sanger.ac.uk). Many studies highlight the remarkable complexity of the TTSS system and AvrA's function. Studies show that AvrA possesses enzyme activities to remove the ubiquitins from IκBα and β-catenin, to transfer acetyl to inhibit JNK activity and to bind with Erk2 and MKK7 [6-9]. Although AvrA is known to regulate diverse bacterial-host interactions, the eukaryotic targets of AvrA are still not completely identified.

Gene expression array technology is a powerful tool that has been used to expand the understanding of host-pathogen interactions. A number of reports have described host transcriptional responses to bacterial infection using microarrays [9-14], but the global physiological function of *Salmonella *effector protein AvrA *in vivo *is unclear. A whole genome approach, combined with bioinformatics assays, is needed to elucidate the *in vivo *genetic responses of the mouse colon to *Salmonella*, and particularly to effector protein AvrA.

In this study, we hypothesized that AvrA interacts with multiple pathways in eukaryotic cells to manipulate the host defense system. A central feature of *S*. Typhimurium pathogenesis is its ability to induce intestinal inflammation [9]. Hence, we specifically examined the gene expression profiles in mouse colon when it responded to pathogenic *Salmonella *stain SL1344 (with AvrA expression) or SB1117 (without AvrA expression). SB1117 is an AvrA mutant strain derived from SL1344. We focused on the intestinal responses to *Salmonella *infection at the early phase (8 hours) and the late phase (4 days). Ingenuity Pathways Analysis (IPA) was used to search for networks of biologically related genes that were co-regulated or differentially regulated in response to SL1344(AvrA+) and SB1117 (AvrA-). The gene expression differences found with the microarray were confirmed using real-time quantitative reverse transcription PCR (qRT-PCR). We identified the eukaryotic cell targets of AvrA and confirmed the eukaryotic cell signaling pathways targeted by bacterial effector protein AvrA. These studies underscore the importance of the *Salmonella *effector AvrA in intestinal-bacterial interactions.

## Methods

### Bacterial strains and growth conditions

*Salmonella *typhimurium wild-type strain SL1344 (WT) and *Salmonella AvrA *mutant strain SB1117 derived from SL1344 (provided by Dr. Galan) [3,9]. Non-agitated microaerophilic bacterial cultures were prepared by inoculating 10 ml of Luria-Bertani broth with 0.01 ml of a stationary phase culture, followed by overnight incubation (~18 h) at 37°C as previously described [15,16].

### Streptomycin pre-treated mouse model

Animal experiments were performed using specific-pathogen-free female C57BL/6 mice (Taconic, Hudson, NY) that were 6-7 weeks old. The protocol was approved by the University of Rochester University Committee on Animal Resources (UCAR). Water and food were withdrawn 4 hours before oral gavage with 7.5 mg/mouse of streptomycin. Afterwards, animals were supplied with water and food ad libitum. Twenty hours after streptomycin treatment, water and food were withdrawn again for 4 hours before the mice were infected with 1 × 10^7 ^CFU of *S. Typhimurium *(100 μl suspension in HBSS) or treated with sterile HBSS (control) by oral gavage as previously described [17]. The wild-type *Salmonella *and AvrA mutant strains were in the same phase of growth. Mice without *Salmonella *infection were set up as the control group (n = 3). At 8 hours and 4 days after infection, mice were sacrificed and tissue samples from the intestinal tracts were removed for analysis, as previously described [17,18]. Three independent biological replicates in every group were performed.

### Sample RNA preparation

Mice were sacrificed at 8 hours and 4 days after *Salmonella *infection, and tissue samples from the intestinal colon mucosa were removed. Total RNAs were isolated using TRIzol reagent (Invitrogen) following the manufacturer's protocol, followed by on-column digestion of DNA using the RNeasy Mini Kit (Qiagen). RNA quantity and quality were assessed with a Beckman Coulter DU 640 Spectrophotometer (Beckman Coulter) and Agilent 2100 Bioanalyzer (Agilent), following the manufacturer's protocols.

### Gene array processing and statistical analysis

The biotinylated single-stranded cDNA was prepared from 100 ng total intact RNA extracted from *Salmonella *infected mouse mucous at 8 hours and 4 days postinfection, or from uninfected mouse control samples. Mouse cDNA was hybridized to the Mouse Gene 1.0 ST array, a microarray chip containing 28,000 sequenced mouse genes (Affymetrix, Santa Clara, CA).

After hybridization, the array was washed and stained with streptavidin-phycoerythrin, and scanned in a proprietary Affymetrix scanner, according to the GeneChip^® ^Whole Transcript Sense Target Labeling Assay manual. The fluorescence values for each feature on the array were measured and recorded. Suite Software (Affymetrix) was used to produce a CEL file. The data were processed with Expression Console (Affymetrix) using the PLIER algorithm. The Array Assist Lite software package was used to generate GC-RMA files (log2 transformed) for each chip. All procedures were performed in triplicate at the Functional Genome Center of the University of Rochester. Fold change was calculated for each strain relative to the uninfected control. Statistical significance (*p *value) was calculated by Student's *t *test, based on the results of three separate experiments. Insignificant genes that changed by less than 1.2 fold were removed from subsequent analysis. The 1.2 cut-off is acceptable in the genomics analysis field [19,20].

### Gene ontology enrichment and pathway analysis

Degree of enrichment for cellular component, biological processes and molecular functions was assessed by the Gene ontology (GO) program [21].

IPA (Ingenuity Systems http://www.ingenuity.com) is a web-based software application tool, which allows for the mapping of gene expression data into relevant pathways based on their functional annotation and known molecular interactions [22-24]. Differential expression analyses between the normal control and *Salmonella*-infected groups were carried out with GeneSifter software.

The IPA program was used mainly for signal transduction pathway analyses and generating pathway figures and tables of related candidate genes. To compare the significant value of the canonical pathway associated with SL1344 and SB1117 infection, we used the Canonical Pathway analysis software package in IPA software. The significance of a given pathway in a dataset is a measurement of the likelihood whether this pathway is associated with the dataset by random chance. IPA software can compare one observation to another. Within a comparison, we could start by comparing the extent to which the significances change from one observation to another. Significance of the canonical pathways was tested by the Fisher Exact test. Data from repeated experiments were clustered within 1.2-fold changes, indicating that the experiments produced reproducible data.

### Hierarchical Cluster analysis

After removing duplicate probe sets, a total of 904 genes showed *p*-value ≤ 0.05 and estimated FC ≥1.2 at least one of the 4 group samples pair-wise comparisons during *S*L1314 and SB1117 infection respectively (SL1344 at 8 hours vs. Control, SL1344 at 4 days vs. Control, SB1117 at 8 hours vs. Control and SB1117 at 4 days vs. Control). This list was used to perform a hierarchical cluster analysis and to construct a heat map using the Gene Cluster 3.0 and tree view software (Stanford University, 2002).

### Real-time quantitative reverse transcriptase PCR (qRT-PCR)

Total RNA was reverse transcribed with oligoDT primer using an Invitrogen SuperScript III kit. The cDNA was subject to qRT-PCR using SYBR Green Supermix (Bio-Rad). A total of 10 differentially-expressed genes in microarray data were chosen for further analysis. Primers of target genes are listed in Additional file 1 Table S1. The amplification conditions were optimized for the MJ research DNA Engine instrument, using melting curve and electrophoresis analysis. The cycling conditions using SYBR green detection were 95°C for 2 min, followed by 40 repetitive cycles at 95°C for 15 s, 58-60°C for 40 s, and 72°C for 30 s. A melting curve analysis was performed from 60°C to 95°C. β-actin was selected as the endogenous control. The threshold cycle (Ct) was determined, i.e. the cycle number at which the fluorescence of the amplified product crosses a specific threshold value in the exponential phase of amplification. Relative quantification of target gene expression was evaluated using the comparative cycle threshold method as previously described by Livak and Schmittgen [25].

## Results and Discussion

### Gene expression in the mouse colon in response to *Salmonella *infection

In this study, we focused on *in vivo *intestinal responses to *Salmonella *AvrA using the SL1344 (AvrA+) and AvrA- strain SB1117. SL1344 is known to constitutively express AvrA protein [3,26,27]. SB1117 lacks AvrA protein expression due to the AvrA mutation derived from SL1344 [3,18,26]. We performed microarray hybridization with RNA from mouse colon mucosa. Biotin-labeled target cDNAs prepared from total RNA extracted were hybridized to the microarray chip containing 28,000 sequenced genes. We selected genes that changed in response to *Salmonella *infection at 8 hours and 4 days time points. Clustering algorithm analysis indicated that the data generated in different arrays at the same time points were tightly clustered (data not shown).

### Cluster analysis

**Figure 1:**
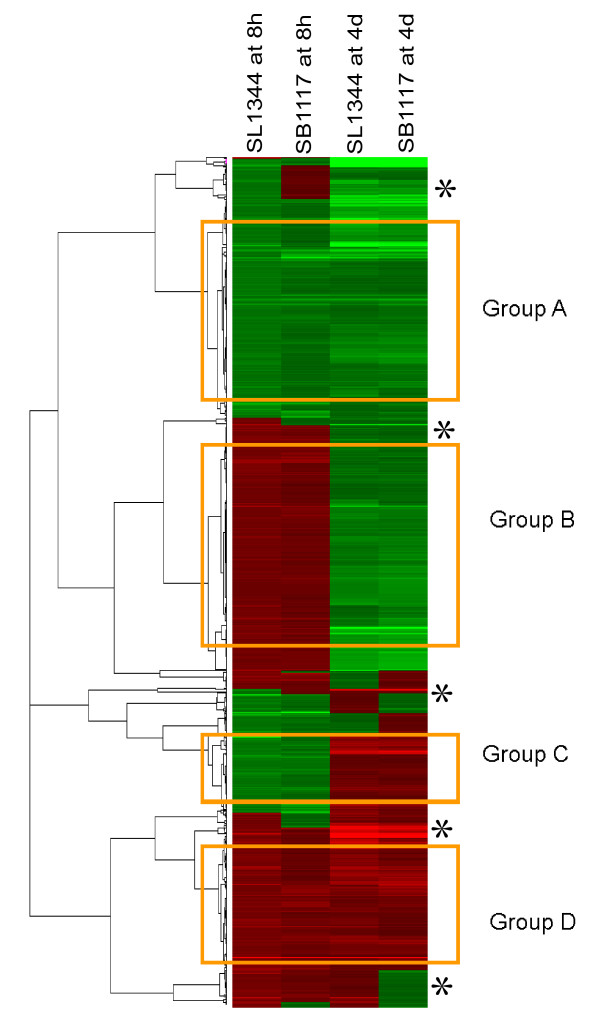
**Hierarchical clustering analysis of 913 genes from Affymetrix array analysis showing differential expression patterns during SL1344 (WT AvrA) infection and SB1117(AvrA-) infection**. A indicates repressed gene cluster at 8 hours and 4 days; B indicates a up-expressed gene cluster at 8 hours but a down-expressed cluster at 4 days; C indicates a down-expressed gene cluster at 8 hours but a up-expressed cluster at 4 days; and D indicates an induced gene cluster at 8 hour and 4 days. Subset group was indicated with*. The heat map was built by using Gene Cluster 3.0 software. Red color represents up-regulation and green shows down-regulation.

In order to obtain a broad overview of the changes in gene expression during SL1344 and SB1117 infection and identify differentially expressed genes clusters between SL1344 infection and SB1117 infection, we generated a heat map using Gene cluster 3.0 for the 913 differentially expressed genes. As shown in Figure 1 overall, SL1344 infection and SB1117 infection showed similar gene expression cluster at 8 hours and 4 days. Four distinct clusters were produced: group A indicated a repressed gene cluster at 8 hours and 4 days; group B indicated an up-expressed gene cluster at 8 hours but a down-expressed cluster at 4 days; group C indicated a down-expressed gene cluster at 8 hours but an up-expressed cluster at 4 days; group D indicated an induced gene cluster at 8 hours and 4 days.

We further identified some subset groups (indicated with *), which suggested that SL1344 and SB1117 infection differentially regulated genes at both the early stage and the late stage. These results indicate that AvrA is involved in altering host responses in the *Salmonella*-intestine interaction *in vivo*.

### Characteristics of differentially expressed genes between the SL1344 and SB1117 infection groups

**Figure 2:**
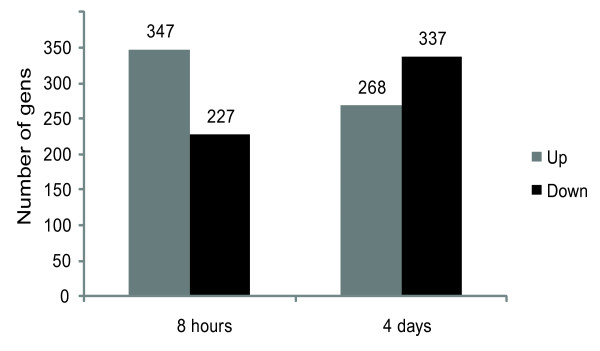
**The number of differentially expressed genes between infection with salmonella, SL1344 (WT, AvrA) and SB1117(AvrA-)**.

Our cluster analysis for the SL1344 (AvrA+) and SB1117 (AvrA-) infection groups have indicated that AvrA expression in the *Salmonella *strains clearly alters the *in vivo *host responses to intestinal infection. In order to get a broad overview of the mouse colon transcriptional changes induced by *Salmonella Typhimurium *SL1344 effector AvrA, fold change in gene expression was calculated for each SL1344 infection group relative to each SB1117 infection group (Figure 2).

In the SL1344 infection group, compared to the SB1117 infection group, at 8 hours post infection, 347 (58%) genes were up-regulated and 227 genes (42%) were down-regulated (Figure 2 and Additional file 2 Table S2, Fold times ≥1.2 times, P ≤ 0.05). In the SL1344 infection group at 4 days, 268 genes (44%) in the group were up-regulated and 337 genes (56%) were down-regulated, compared to the SB1117 infection group (Figure 2 and Additional file 3 Table S3, Fold times ≥1.2 times, P ≤ 0.05). The majority of the genes that were differentially expressed between groups showed moderate alterations in expression of 1.2 to 2.0 folds (Additional file 2 Table S2 and Additional file 3 Table S3). Overall, the results indicate that AvrA protein by TTSS must be responsible for the induction and repression of *in vivo *transcriptional reprogramming of the host cells in intestinal infection (Figure 2).

To investigate co-regulated biological processes by AvrA during the early and late stages of SL1344 infection, we searched co-differentially expressed genes during the infection by using Filter datasets package of IPA software: *DNAH3 *was the only annotated up-regulated gene at both 8 hours and 4 days post infection; it is involved in nucleotide binding, ATP binding and microtubule motor activity. *GRK5 *(G protein-coupled receptor kinase 5) was the only annotated down-expressed gene at both 8 hours and 4 days post infection. *GRK5 *plays a positive role in Crohn's disease [28]. *Salmonella *infection increases the risk of inflammatory bowel diseases (IBD) including Crohn's disease [29]. It is interesting to explore the potential role of AvrA in the *Salmonella*-related IBD. *Notch3 *was annotated with up-regulation at 8 hours post infection, but showed down-expression at 4 days post infection. *MS4A7 *was down-expressed at 8 hours post infection and up-expressed at 4 days post infection. These unique co-regulated genes suggest that AvrA function is differentially regulated in host cells in association with infection time.

### Validation of microarray findings with real-time PCR

**Figure 3:**
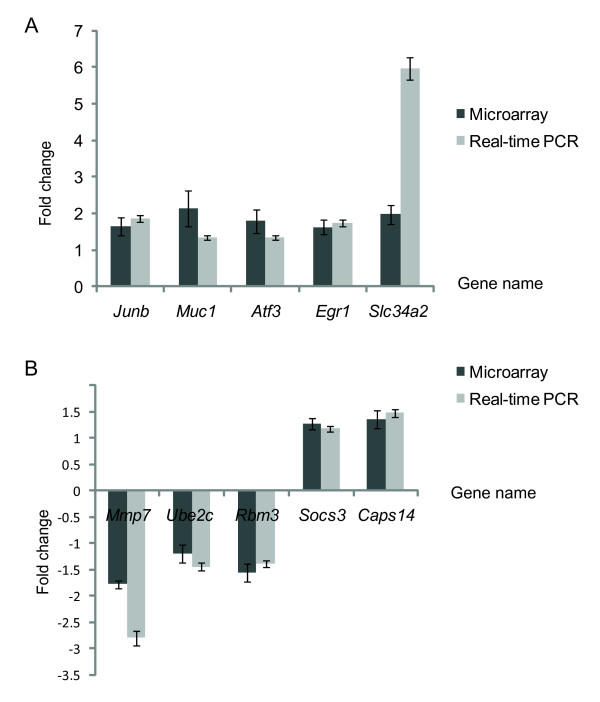
**Real-time PCR analysis and Microarray Comparison**. A: real-time PCR analysis and microarray comparison at the early stage of Infection. B: real-time PCR analysis and microarray comparison at the late stage of infection.

To validate microarray results, we selected 10 differentially expressed genes between SL1344 infection group and SB1117 infection group for qRT-PCR. All of qRT-PCR analyses were performed in samples previously used for the microarray experiments (Figure 3). Figure 3A and Figure 3B showed the fold times in gene expression in microarray data and real-time PCR measurements at the early stage and the late stage of infection respectively. The gene expression changes measured by qRT-PCR were in agreement with microarray data.

The Pearson correction coefficient between the qRT-PCR and microarray data was 0.836. Therefore, the microarray provided a reliable comparison of gene expression in mouse colon mucous sample from salmonella SL1344 and SB1117 infection at 8 hours and 4 days.

### Gene Ontology (GO) terms enrichment analysis for genes differentially expressed between the SL1344 and SB1117 infection groups

The analysis of enriched GO terms could aid in interpreting the dominant functions controlled by differentially expressed genes. To further address the potential contribution of AvrA to the *S*. typhimurium SP-I TTSS-mediated stimulation of transcriptional response in mouse intestine, we evaluated the biological processes for these differentially expressed genes, using the GO term enrichment on-line analysis tool, GOEAST (Gene Ontology Enrichment Analysis Software Toolkit) [21]. Table 1, 2, 3, 4 lists important Gene Ontologies with P-values less than 0.05.

**Table 1:** List of biologic process for the up-expressed genes in SL1344 infection group relative to that of SB1117 infection group at 8 hr

GO ID	Term	No. of genes
GO:0007154	cell communication	71
GO:0007165	signal transduction	64

**Table 2:** List of biologic process for the down-expressed genes in SL1344 infection group relative to that of SB1117 infection group at 8 hr

GO ID	Term	No. of genes
GO:0006996	organelle organization	20
GO:0007049	cell cycle	14
GO:0051276	chromosome organization	10
GO:0006334	nucleosome assembly	9
GO:0031497	chromatin assembly and disassembly	9
GO:0034728	nucleosome organization	9
GO:0065004	protein-DNA complex assembly	9
GO:0006323	DNA packaging	9
GO:0034622	cellular macromolecular complex assembly	9

**Table 3:** List of biologic process for the up-expressed genes in SL1344 infection group relative to that of SB1117 infection group at 4 day s

GO ID	Term	No. of genes
GO:0065007	biological regulation	70
GO:0050794	regulation of cellular process	66
GO:0032501	multicellular organismal process	47
GO:0007165	signal transduction	45
GO:0007154	cell communication	45
GO:0007166	cell surface receptor linked signal transduction	38
GO:0042221	response to chemical stimulus	14
GO:0006915	apoptosis	10
GO:0008219	cell death	10

**Table 4:** List of biologic process for the down-expressed genes in SL1344 infection group relative to that of SB1117 infection group at 4 days

GO ID	Term	No. of genes
GO:0003008	system process	39
GO:0050877	neurological system process	37
GO:0007186	G-protein coupled receptor protein signaling pathway	35
GO:0007608	sensory perception of smell	27
GO:0007606	sensory perception of chemical stimulus	27
GO:0007268	synaptic transmission	7

In 347 up-regulated genes in the SL1344 infection group relative to SB1117 infection group at 8 hours (Table 1), 230 transcripts were assigned specific GO terms. GOEAST analysis showed that most of these genes participated in cell communication (71 genes) and signal transduction (64 genes).

Shown in Table 2, 227 genes were down-regulated in the SL1344 infected group relative to SB1117 infection group at 8 hours. We found that 174 transcripts were assigned specific GO terms. Of these transcripts, 76.6% were annotated as being involved in biological processes, and a significant number of transcripts were assigned known functions in organelle organization (20 genes), cell cycle (14 genes), chromosome organization (10 genes), chromatin assembly and disassembly (9 genes), nucleosome organization (9 genes), protein-DNA complex assembly (9 genes), DNA packaging (9 genes), and cellular macromolecular complex assembly (9 genes). Annotation showed that many of the genes belong to the centromere protein and the histone family protein. This result indicates that most of biological processes down-regulated by AvrA relate to nuclear function.

**Figure 4:**
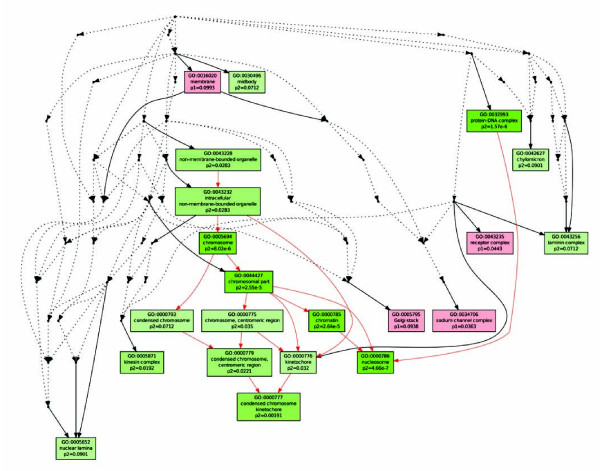
**Graphical output of Multi-GOEAST cellular component analysis results for genes differentially expressed by SL1344 and SB1117 infected mouse colon at 8 hours**. Red Boxes represent enriched GO terms only found in up-regulated genes in the SL1344 vs SB1117 infection groups, and green boxes represent enriched GO terms only found in down-regulated genes in SB1344 vs SB1117 infection groups. The saturation degrees of all colors represent the significance of enrichment for corresponding GO terms. Arrows represent connections between different GO terms. Red arrows represent relationships between two enriched GO terms, black solid arrows represent relationships between enriched and unenriched terms and black dashed arrows represent relationships between two unenriched GO terms.

In order to confirm the analysis results, we compared the cellular component of ontology for the two groups using the Multi-GOEAST analysis tool. As shown in Figure 4 all of the down-regulated GO terms are associated with the nucleus (green box), whereas up-regulated processes were associated with membrane and cytoplasm (red box). We observed a significant GO annotation of condensed chromosome/nucleosome related activity (red arrow in Figure 4). Therefore, we speculate that chromosome/nucleosome process activities are strongly affected by AvrA at 8 hours post infection by SL1344.

In Table 3, 268 genes were up-regulated in the SL1344 vs SB1117 infection groups at 4 days. Among them, 134 transcripts were assigned specific GO terms. A significant number of transcripts were assigned known functions in biological regulation (70 genes), regulation of cellular process (67 genes), multicellular organismal process (47 genes), signal transduction (45 genes) and apoptosis (10 genes). An interesting result was that a total of 25 differentially expressed olfactory receptor family members participated in all of the biological processes except for apoptosis (Table 3).

In the SL1344 vs SB1117 infection group at 4 days, 337 genes were down-regulated genes (Table 4). Of these gene, 201 transcripts were assigned specific GO terms, and a significant number of transcripts were assigned known functions in system process regulation (39 genes), neurological system processes (37 genes), and G protein-coupled receptor protein signaling pathway (35 genes). These biological processes may underlie the physiological deficits of bacterial infection by inducing a decline in gene transcription.

The ontology of the cellular component for down-regulated and up-regulated genes showed that most of molecular activity occurred in the cell membrane at 4 days post infection (data not shown).

### AvrA targeted specific pathway and network analysis

An over-representation of a specific biological process does not indicate whether the process in question is being stimulated or repressed overall. We used IPA software to further investigate over- or under-represented functional activities of AvrA, specifically within the up-regulated and down-regulated genes, at the stage of infection at 8 hours and 4 days. We focused on the ingenuity canonical pathways and addressed the differentially up-regulated genes between the SL1344 vs SB1117 infection groups at 8 hours and 4 days post infection (Table 5 and Table 6).

**Table 5:** Target pathway of up-regulated Genes in SL1344 vs SB1117 infection groups at 8 hours.

Ingenuity Canonical Pathways	Up-regulated	Molecules
Cholecystokinin/Gastrin mediated Signaling	8/104 (8%)	FOS, RHOV, JUN, RHOB, RHOD, IL1RN, RHOC, IL1F8
MIF Regulation of Innate Immunity	3/46 (7%)	FOS, LY96, JUN
Complement System	2/36 (6%)	C3, C1S
FAK Signaling	6/98 (6%)	CAPN5, ARHGAP26, CAPN9, CAPN2, ITGA3, ACTC1
HMGB1 Signaling	6/98 (6%)	FOS, RHOV, JUN, RHOB, RHOD, RHOC
IL-10 Signaling	4/70 (6%)	FOS, JUN, IL1RN, IL1F8
Integrin	111/200(6%)	CAPN5, RHOV, ARHGAP26, RHOB, RHOD, ITGA11, RHOC, CAPN9, CAPN2, ITGA3, ACTC1
	7/112 (6%)	PLCZ1, RHOV, RHOB, RHOD, RHOC, PLCL2, CASP14
Toll-like Receptor Signaling	3/54 (6%)	FOS, LY96, JUN
B Cell A ctivating Factor Signaling	2/44 (5%)	FOS, JUN
Coagulation System	2/37 (5%)	FGA, F3
CXCR4 Signaling	8/168 (5%)	FOS, RHOV, JUN, RHOB, RHOD, RHOC, EGR1, GNG5
IL-2 Signaling	3/58 (5%)	FOS, JUN, SYK
ILK Signaling	10/187(5%)	MUC1, FOS, RHOV, JUN, RHOB, RHOD, RHOC, PPM1J, ACTC1, IRS3
Phototransduction Pathway	3/65 (5%)	OPN3, GRK1, PDE6H
Production of Nitric Oxide and reactive Oxygen Species in Macrophages	10/185 (5%)	FOS, RHOV, JUN, RHOB, PPP1R14 D, RHOD, RHOC, PPM1J, HOXA10, SPI1
Regulation o f Actin based	5/93 (5%)	RHOV, RHOB, RHOD,
Motility by Rho		RHOC, ACTC1
Role of Cytokines in Mediating Communication between Immune Cells	3/57 (5%)	IL1RN, IFNA5, IL1F8
Role of Pattern Recognition Receptors in Recognition of Bacteria and Viruses	4/86 (5%)	IRF4, C3, SYK, IFNA5
	4/114 (4%)	PLCZ1, FOS, JUN, PLCL2

**Table 6:** Target pathway of up-regulated genes in SL1344 vs SB1117 infection groups at 4 days.

Ingenuity Canonical Pathways	Up-regulated	Molecules
Role of Cytokines in Mediating Communication between Immune Cells	3/57 (5%)	IFNG, IFNA7, IL3 HS6ST1, CYP3A4, 2810007J24RIK,
LPS/IL-1 Mediated Inhibi tion of RXR f unction	5/215 (2%)	IL4I1, ABCC4
Interferon Signaling	2/30 (7%)	IFNG, IRF1
Retinoic acid Mediated Apoptosis Signaling	2/44 (5%)	CFLAR, IRF1
IL-12 Signaling and Pro duction in m acrophages	3/134 (2%)	IFNG, IF NA7, IRF1
Gα12/13 Signaling	3/126 (2%)	BTK, F2RL2, MEF2D
Calcium -induced T Lymphocyte Apoptosis	2/65 (3%)	MEF2 D, ORAI1
Prolactin Signaling	2/75 (3%)	SOCS3, IRF1
VDR/RXR Activation	2/80 (3%)	IFNG, RUNX2
Acute Phase Response Signaling	3/178 (2%)	SOCS3, TF, IL6R
Role of NFAT in Regulation of the Immune response	3/195 (2%)	BTK, MEF2 D, ORAI1
Communication between Innate and adaptive Immune Cells	2/90 (2%)	IFNG, IL3
Fc Epsilon RI Signaling	2/103 (2%)	BTK, IL3
Natural Killer Cell Signaling	2/112 (2%)	KLRK1, HCST
IL-22 Signaling	1/28 (4%)	SOCS3
IL-15 Production	1/31 (3%)	IRF1
p70S6K Signaling	2/131 (2%)	BTK, F2RL2
IL-9 Signaling	1/37 (3%)	SOCS3
JAK/Stat Signaling	1/64 (2%)	SOCS3

At 8 hours post *Salmonella *infection, shown in Table 5 up-expression of the Ras homolog gene family, *RhoB, RhoC, RhoD *and *RhoU*, targeted a total of 7 pathways: cholecystokinin/gastrin-mediated signaling; CXCR4 signaling; High-mobility group box 1(HMGB1) signaling; ILK signaling; integrin signaling; phospholipase C signaling; production of Nitric Oxide, and reactive oxygen species in macrophages, and regulation of actin-based motility by Rho. We found that these pathways are associated with the following functions: cellular assembly and organization, cell to cell signaling and interaction, and infectious diseases.

Furthermore, we found that the up-regulated genes *Fas *and *Jun*, as transcription regulators, co-targeted many of pathways which are implicated as regulators of the stress response (production of Nitric Oxide and Reactive Oxygen Species in Macrophages pathway, IL-2 Signaling pathway, Toll-like Receptor Signaling, and CXCR4 Signaling pathway), inflammation (HMG1 pathway), proliferation (Cholecystokinin/Gastrin-mediated Signaling) and cell apoptosis (14-3-3 mediated signaling B Cell Activating Factor Signaling).

**Figure 5:**
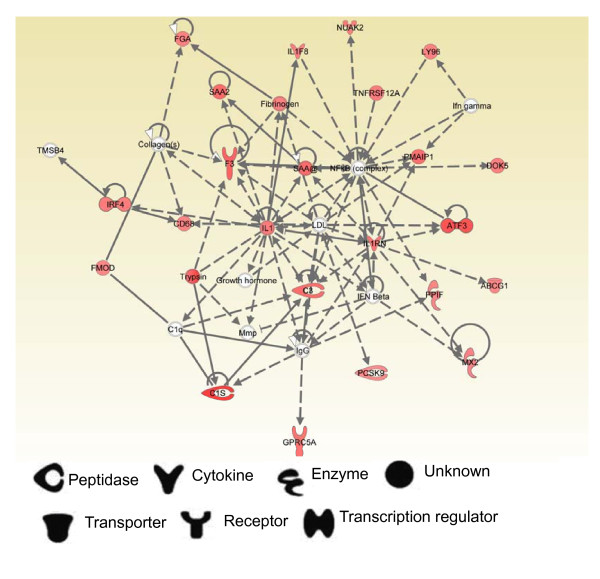
**Ingenuity pathway Analysis network 1 depicting relationships among up-regulated genes in SB300 infection group relative to that of SB1117 infection group at 8 hours**. Intensity of the red color indicates the degree of up-regulation. Nodes are displayed using various shapes that represent the functional class of the gene product. Edges are displayed with various labels that describe the nature of relationship between the nodes: ___ represents direct relationship; ----- represents indirect relationship; → represents acts on.

To clarify AvrA function in interactions between up-regulated genes, we examined gene networks using IPA. As shown in Figure 5 this network presented IL1RN, NF-κB, and IL1 in central positions and corrected the following functions: Cellular assembly and organization, infectious disease, and tissue morphology. Based on the Ingenuity Pathway Knowledge base, around the NF-κB central position, IL1F8, IFNA and IL1RA decrease NF-κB activation, whereas LY96, TNFRSF12A, SAA2, and Fibrinogen increase NF-κB activation. This result showed that AvrA is involved in regulation of NF-κB activation. However, AvrA's role in modulating the NF-κB activity may depend on a complex regulation network.

**Figure 6:**
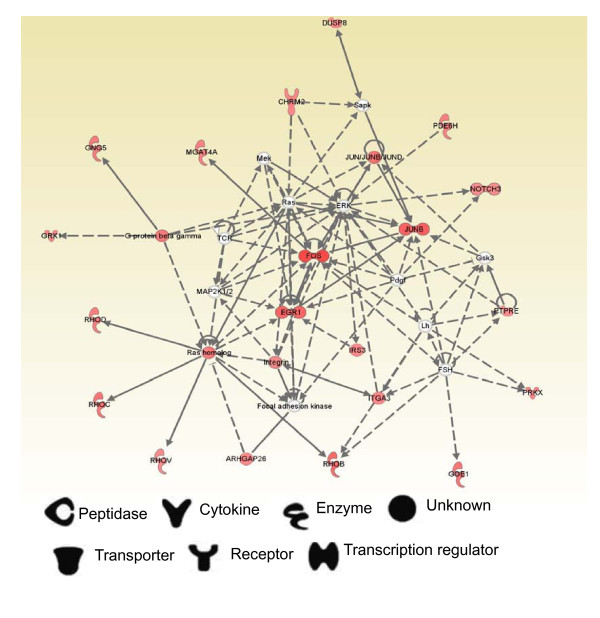
**Ingenuity Pathway Analysis Network 2 depicting relationship among up-regulation Genes in SL1344 vs SB1117 infection groups at 8 hours**. Intensity of the red color indicates the degree of up-regulation. Nodes are displayed using various shapes that represent the functional class of the gene product. Edges are displayed with various labels that describe the nature of relationship between the nodes: ___ represents direct relationship, ----- represents indirect relationship → represents acts on.

As shown in Figure 6 the network also showed the relevance of the Ras homolog, EGR1 group, Fas group and Jun group. In mouse M1 cell lines, EGR1 protein increases expression of mouse *Junb *mRNA [30]. The *Salmonella *Typhimurium type III Secretion effectors, SopE, SopE2 and SopB, stimulate Rho-family GTPase signaling [31,32] and innate immune responses [33,34]. Our study demonstrate that AvrA stabilizes the tight junction structure and protein expression *in vitro *and *in vivo *[35]. Studies on AvrA demonstrated that AvrA reverses the activation of specific signaling pathways induced by effectors delivered by *S*. Typhimurium *via *the same TTSS [9]. Hence, the AvrA may have opposite effects on Rho-family GTPase, whereas the other *Salmonella *effectors stimulate Rho-family GTPase signaling.

Down-expressed genes in SL1344 vs SB1117 infection groups at 8 hours targeted mainly nuclear receptor signaling related pathway, such as PXR/RXR Activation, FXR/RXR Activation, and LPS/IL-1 Mediated Inhibition of RXR Function (Additional file 4 Table S4). The three pathways were co-targeted by the protein product of three genes, *Cyp2c8 (*Cytochrome P_450_2C8), *Aldha1 *(Aldehyde dehydrogenase 1 family, member A1), and *Prkag2 *(5'-AMP-activated protein kinase subunit gamma-2). We also observed decreased expression of the gene for *Fancd2 *in the SL1344 infection group relative to SB1117 infection group. This protein is monoubiquinated in response to DNA damage, resulting in its localization to nuclear foci with other proteins (BRCA1 and BRCA2) involved in homology-directed DNA repair [36-38]. In other words, the down-regulation of *Fancd2 *in the SL1344 infection group relative to the control group implies that AvrA protects from DNA damage at the early stage of SL1344 infection. We also found that *Socs2*, which encodes a member of suppressors of cytokine signaling [39], is down-regulated in the SL1344 vs the SB1117 infection group. The Socs2 protein interacts with the cytoplasmic domain of insulin-like growth factor 1 receptor (IGF1R), and thus regulating IGF1R mediated cell signaling [39].

In addition, as shown in Additional file 3 Table S3, *Socs2 *also targeted JAK pathway signal transduction adaptor activity and participated in regulation of cell growth and anti-apoptosis. Because Socs2 is a negative regulator of cytokine signal transduction that inhibits the JAK/STAT pathway [40,41], the increased levels of the genes in the SL1117 infection group relative to control and SL1344 infection group may help to explain AvrA's proliferation role in activating JAK/STAT pathway at the early stage of SL1344 infection.

At 4 days post *Salmonella *infection, 5 up-regulated expressed genes in SL1344 infection group, compared to SB1117 infection group, overlap with a series of canonical pathways (Table 6): *Ifng, Irf1, Btk, Mef2 d*, and *Socs3*. These pathways have been associated with the following functions: cellular movement, the hematological system, cell proliferation and the hematopoiesis. Interferon-gamma *(*IFNG) is a cytokine critical for innate and adaptive immunity against viral and intracellular bacterial infections and for tumor control [42,43]. This result indicated that at the later stage of *Salmonella *infection AvrA may be involved in regulation of aberrant IFNG expression, which is associated with a number of autoinflammatory and autoimmune diseases.

We observed that another suppressor of cytokine signaling, *Socs3*, is up-regulated in the SL1344 vs. SB1117 infection groups at 4 days postinfection. This result contrasts with the down-expressed status of *Socs2 *at 8 hours. As shown in Table 6 the expression of *Socs3 *through the JAK/STAT pathway negatively regulates cytokine signaling, e.g., signaling of rolactin, acute phase response, IL-9, and IL-22. We found that these pathways are related to cell death; cellular growth and proliferation; as well as gastrointestinal and inflammatory disease. This finding suggests a possible role for AvrA that affects the above functions and diseases through regulation of cytokine signaling.

Down-expressed genes in the SL1344 vs. the SB1117 infection groups at 4 days targeted mainly metabolic related pathways, such as aminophosphonate, histideine and cysteine metabolism (Additional file 5 Table S5). The protein product of Prmt5, which is the protein arginine methyltransferase 5 involved in protein modification, targets these three pathways. As shown in Table S5, *Casq1*, *Chrna4*, and *Ryrs *are related to calcium signaling, and they are down-regulated in SL1344 vs. the SB11117 infection groups, but showed almost unchanged expression in the SL1344 infection group relative to the control. This result implies that AvrA negatively regulates calcium signaling in the late stage of SL1344 infection.

### AvrA function analysis during the time course of SL1344

**Figure 7:**
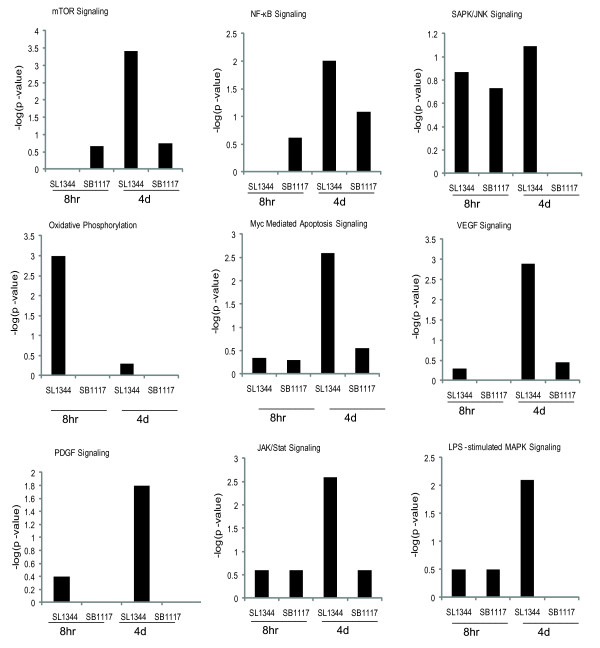
**Canonical pathways identified by IPA associated with SL1344 and SB1117 responsive genes**.

We further used the canonical pathway analysis software package in IPA software to determine whether and to what extent a given pathway is affected by the bacteria effector AvrA. We found many pathways with different signaling responses during the early and late stage of SL1344 and SB1117 infection. Figure 7 lists the nine representative pathways yielded by this analysis.

The mTOR signaling, Myc-mediated cell apoptosis signaling, PDGF, VEGF, JAK-STAT, and LPS-stimulated MAPK signaling were most significant at the stage of SL1344 infection compared to SB1117 infection after 4 days (Figure 7). However, these pathways were less significant at the early stage of SL1344 and SB1117 infection (8 hours). Hence, this analysis confirmed the functional performance of AvrA in late stage of SL1344 infection. We also found that these above pathways were closely related to biological processes of cell apoptosis. These observations are consistent with the signaling transduction studied on AvrA in anti-apoptosis [7,8]. Therefore, AvrA plays an essential role in anti-apoptosis by regulating multiple signaling pathways *in vivo*.

Unlike the above pathways, oxidative phosphorylation showed the most significant signaling at the early stage of SL1344 vs. SB1117 infection. Our results also showed that AvrA had no important function in regulating oxidative phosphorylation pathway at the late stage of infection (Figure 7 Oxidative phosphorylation).

NF-κB signaling is a key player in inflammation [44,45]. We found that NF-κB was less significant in SL1344 vs. SB1117 infection at early stage of infection, but at the late stage of SL1344 infection, NF-κB signaling showed higher significance than that of SB1117 infection (Figure 7). Post-transcriptional study demonstrated that AvrA inhibits the NF-κB activity though stabilizing the inhibitor of NF-κB, IκBα [6,8]. Overall, this result implies that AvrA suppressed the NF-κB activity at the early stage of SL1344 infection and has a different regulatory role at the late stage.

In contrast, the significance values of SAPK/JNK signaling were low at late stages of SB1117 infection, which suggest that SL1117 infection is not associated with the SAPK/JNK pathway at the late stage of Infection.

### AvrA regulation of the mTOR, NF-κB, JNK, and oxidative phosphorylation signaling pathways *in vivo*

**Figure 8:**
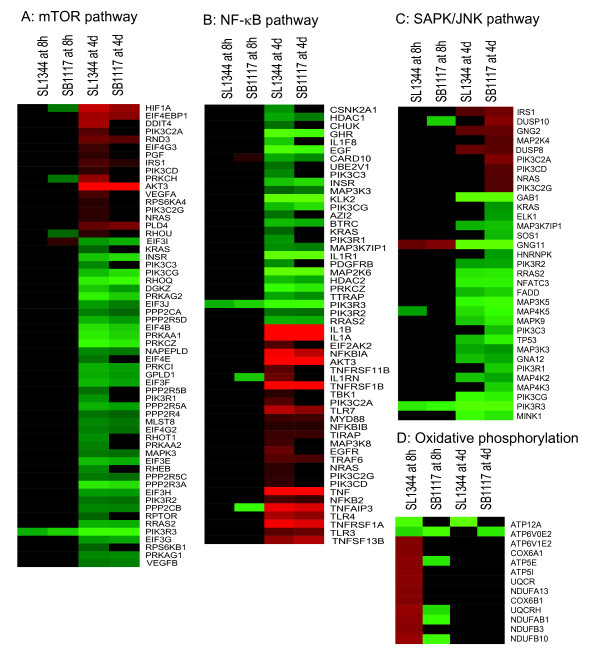
**Heat maps of *Salmonella*-responses to gene expression changes involved in four signaling transduction pathways**. A: mTOR signaling; B: NF-κB pathway C:SAPK/JNK signaling; D: Oxidative phosphorylation. Red denotes up-regulation; Green denotes down-regulated genes, black denotes unchanged or P-value > 0.05 in three replicate experiments.

It is possible that the genes that underlie the biology of a pathway could be different from one observation to another, even if the significant values remain unchanged. To evaluate this possibility, we performed a cross-analysis comparison of the genes associated with a given pathway during the early and late stages of SL1344 and SB1117 infection. To further analyze the AvrA regulation of the mTOR, NF-κB, JNK, and oxidative phosphorylation signaling pathways *in vivo*, we generated heat maps to investigate the associated genes in these pathways (Figure 8A-D).

As shown in Figure 8A, many genes of mTOR pathway play a role in cell proliferation, migration, apoptosis, differentiation, growth, and cell death. *VEGFA*, *PIK3C2A*, *PIK3CD*, *PIK3C2G*, and *PRKCH *showed up-regulation in the SL1344 infection group at the late stage of infection, whereas in the SB1117 infection group, the expression of these genes showed no significant change. These data indicated that AvrA is involved in the mTOR signaling pathway, thus playing a role in proliferation and apoptosis.

Figure 8B showed that *Card10 *was up-regulated at the early stage of SB1117 infection, but not at the early stage of SL1344 infection. The Card10 protein is a caspase recruitment domain/membrane-associated guanylate kinase family that interacts with BCL10 and activates NF-κB-inducing kinase activity [46]. Hence, the result showed that AvrA may inhibit NF-κB activation at the early stage of SL1344 infection relative to SB1117 infection. However, at the late stage of infection, many genes were differentially expressed between the SL1344 vs. SB1117 infection groups. These genes including down-regulated *KRAS, PIK3R1, PDGFRB, CHR, CHUK *and *CSNKIA1*, as well as up-regulated genes *TLR4, TLR3 and TLR7, EIF2AK2, TBk1*, and *PIK3C2A*. Because the listed genes are involved in both negative and positive regulation for NF-κB activation respectively, AvrA function for the NF-κB pathway was more complex at the late stage of SL1344 infection.

We observed that *Dusp10 *is up-regulated at 8 hours post SB1117 infection, but no expression change was observed at 8 hours post SL1344 infection (Figure 8C). Because DUSP10 negatively regulates JNK and p38MAPK [47,48], we reasoned that AvrA may stabilize DUSP10 expression to inhibit activation of JNK pathway at the early stage of SL1344 infection. However, more up-regulated and down-regulated genes that participate in response to the MAPKK signaling cascade are involved at the late stage of both SL1344 and SB1117 infection, there is no clear evidence that AvrA functions differently in the SAPK/JNK pathway at the late stage.

Figure 8D listed genes involved with oxidative phosphorylation at 8 hours post SL1344 infection, compared to the same time post SB11117 infection. These genes included ATP synthase family members (*ATP5E*, *ATP5I*, and *ATP6V1*), cytochrome C oxidase family members (*Cox6A1 *and *Cox6B1*), NADH dehydrogenase family members (*NDUFA1, NDUFAB, NDUFB3, NDUDB1*and *NDUFS5*), and Ubiquinol-cytochrome-c reductase family members (*URCR *and *URCARH*). The oxidative phosphorylation pathway covers a series of oxygen and redox reactions within mitochondria. AvrA may be involved in regulation of mitochondrial function at the early stage of infection.

### Comparison the role for AvrA in microarray analysis with previous study

As shown in Table 7 several previous studies have reported that AvrA functions in these pathways, including JNK, NF-κB, p53, β-catenin, and tight-junction signaling. Similar to the previous results, our microarray analysis for AvrA role at the early stage of infection further reveal that AvrA can lead to gene expression changes of JNK and NF-κB pathway. Moreover, our study extended the understanding of AvrA in inhibiting the JNK and NF-κB pathways.

**Table 7:** Summary of publications regarding the role for Salmonella AvrA in monolayers, drosophila, and mouse models.

Models	Pathways	References
Monolayers	Tight-junction pathway	Liao et al., PLoS One. 2008 3(6):e236
	Activated β-catenin pathway	Sun et al., Am J Physiol Gastrointest Liver Physiol. 2004 287(1):G220-7
	Inhibited NF-κB pathway	Ye et al., Am J Pathol. 2007 171(3):882-92
	Inhibited NF-κB pathway	Collier-Hyams et al., J Immunol. 2002 169(6):2846-50
	Inhibited JNK pathway	Du and Galan, PLoS Pathog. 20095(9): e1000595
	Inhibited JNK pathway	Jones et al, Cell Host Microbe. 2008 3(4):233-44
*Drosophila*	Inhibited JNK, NF-κB pathway	Jones et al, Cell Host Microbe. 2008 3(4):233-44
Mouse	Inhibited JNK, NF-κB pathway	Jones et al, Cell Host Microbe. 2008 3(4):233-44
	Inhibited NF-κB pathway	Ye et al., Am J Pathol. 2007 171(3):882-92
	Activated P53 pathway	Wu et al., Am J Physiol Gastrointest Liver Physiol. 2010 298(5):G784-94.
	Tight-junction pathway	Liao et al., PLoS One. 2008 Jun 4;3(6):e236
	Activated β-catenin pathway β	Ye et al., Am J Pathol. 2007 Sep;171(3):882-92
	Activated Wnt/β-catenin Pathway	Liu et al., FEBS Letter, 2010 584(5):911-916

However, the microarray study has its limitations to identify the post-transcriptional and posttransductional behavior of the differentially expressed genes. This method may also have statistical error. We have demonstrated that *Salmonella *effector AvrA can activate β*-*catenin pathway through deubiquitination [8]. However, the activated pathway was not reveled in the current analysis. Hence, further studies combined genomic and proteomic are necessary to explore further details of AvrA function in interplaying with host cell.

## Conclusion

In this study, we have used DNA microarrays to define the molecular regulators of intestinal signaling and host defense expressed in adult C57Bl/6 female mice during the early and late phases of infection with virulent SL1344 (AvrA+) or isogenic AvrA-*Salmonella *strains. We identified pathways, such as mTOR signaling, oxidative phosphorylation, NF-κB, VEGF, JAK-STAT, and MAPK signaling regulated by AvrA *in vivo*, which are associated with inflammation, anti-apoptosis and proliferation. At the early stage of *Salmonella *infection, down-regulated genes in the SL1344 vs SB1117 infection groups mainly targeted pathways related to nuclear signaling and up-regulated genes in the SL1344 vs SB1117 infection groups mainly targeted oxidative phosphorylation. At the late stage of *Salmonella *infection, AvrA inhibits Interferon-gamma responses. Both early and late phases of the host response exhibit remarkable specificity for the AvrA+ strain in intestine. These results provide new insights into the molecular cascade, which is mobilized to combat *Salmonella*-associated intestinal infection *in vivo*.

Our *in vivo *data indicated that the status of AvrA in *Salmonella *strains may alter the strains' ability to induce host responses, especially in the intestinal mucosa response. Our recent study on AvrA further demonstrates that AvrA enhances intestinal proliferation *in vivo *[18,49]. Although the exact function and mechanism of AvrA is not entirely clear, it is known that AvrA is a multifunctional protease that influences eukaryotic cell pathways that utilize ubiquitin and acetylation, thus inhibiting apoptosis and promoting intestinal proliferation [7,8].

Our microarray data analysis indicated that NF-κB is one of the top-10 signaling pathways targeted by AvrA *in vivo*. A recent study showed that AvrA inhibits the *Salmonella*-induced JNK pathway but showed a very weak inhibition of the NF-κB signaling [9]. The different findings about the AvrA's regulation of the NF-κB pathway may be due to the different experimental system used and different stage post infection. Because the NF-κB is centrally involved of inflammatory networking, other functions of AvrA may indirectly influence the NF-κB activity [35,50].

AvrA status affects levels of expression of the other effector proteins in *Salmonella *([51] and unpublished data). Stimulation of inflammation by bacterial effectors is crucial for *Salmonella *to grow in the intestine [52,53]. However, un-controlled inflammation is harmful to the host and eventually damages the niche involved *Salmonella *growth. AvrA plays a role opposite to that of the other known effectors by inhibiting the inflammatory responses in intestine. Hence, one could argue that AvrA's role in inhibiting inflammation allows the pathogen to survive well in the host, thus establishing a mutually beneficial relationship.

Our current study investigated gene expression at the mRNA level in response to AvrA. Posttranscriptional modification by AvrA cannot be identified by DNA array analysis. Study using Western blot and other protein assay methods will provide further insights into the AvrA's regulation of eukaryotic proteins in intestine.

Taken together, our findings show that AvrA specifically inhibits inflammatory responses and promotes proliferation *in vivo*. It is important to understand how AvrA works *in vivo *because of the *Salmonella *problems and the bioweapon threat of bacterial toxins. We believe that studies on the action of bacterial effectors will uncover new facets of bacterial-host interaction that may lead to the development of new therapeutic drugs or vaccines against important human pathogens.

## Abbreviations

CXCR4: CXC chemokine receptor 4, also known as fusin; EGF: Epidermal growth factor; ErK: extracellular signal-regulated kinases; GO: Gene ontology; GSK-3: glycogen synthase kinase 3; HMGB1: High-mobility group box 1; IBD: inflammatory bowel diseases; ICAM: intercellular adhesion molecule; IFNA: Interferon-alpha; IFNG: Interferon-gamma; IκBα: Inhibitor of NF-κB; IKK: IκB kinase; IPA: Ingenuity Pathways Analysis; IL1RA: IL-1 receptor antagonist; IGF1R: insulin-like growth factor 1 receptor; JAK-STAT:Janus kinases-Signal Transducers and Activators of Transcription protein JNK: JUN-NH_2_-terminal kinase; LPS: Lipopolysaccharides; MAPK: mitogen-activated protein kinase; MKK7: Mitogen-activated protein kinase kinase 7, also known as MAP2K7; MCP-1: monocyte chemoattractant protein 1; mTOR: mammalian target of rapamycin; NF-κB: Nuclear factor κB; PDGF: platelet-derived growth factors; PXR: pregnane × receptor, or NR1I2 (nuclear receptor subfamily 1, group I, member 2) RXR: Retinoid × receptor; SAKP: Stress-activated protein kinase; TLR: Toll-like receptor; TNF: Tumor necrosis factor; TNFRSF12A: Tumor necrosis factor receptor superfamily member 12A; TTSS: Type Three Secretion System; STRAF6: tumor necrosis factor receptor-associated factor-6; VEGF: vascular endothelial growth factor; YopJ: *Yersinia *outer protein J

## Authors' contributions

XL: participated in experimental design, animal experiment, preparation of RNA sample, real-time PCR, western blot and immunofluorescence analysis, acquisition of data, analysis and interpretation of data, carried out bioinformatics analysis, and drafted table, figure and the manuscript.

RL: participated in experimental design, analysis and interpretation of data, real-time PCR analysis, drafted tables and figures, and carried out animal experiments.

YX: participated in interpretation of data, performed statistical analysis, and edited the manuscript for important intellectual content.

SW: participated in experimental design, technical support, animal experiments, analysis and interpretation of data.

JS: participated in study concept and design, acquisition of data, analysis and interpretation of data, material support, writing and critical revision of the manuscript for critical intellectual content, obtained funding, and supervised study.

All authors read and approved the final manuscript.

## Supplementary Material

Additional file 1 **Table S1. Primer sequence for qRT-PCR**. Listing all primer sequences used in qRT-PCR (PDF file). PCR data were shown in Figure 3. Click here for file

Additional file 2 **Table S2. Differentially expressed genes between the SL1344 infection and the SB1117 infection at early stage**. The list of differentially expressed genes between the SL1344 infection and the SB1117 infection at 8 hours post-infection (P ≤ 0.05 with fold change≥1.2 or ≤-1.2). Click here for file

Additional file 3 **Table S3. Differentially expressed genes between the SL1344 infection and the SB1117 infection at late stage**. The list of differentially expressed genes between the SL1344 infection and the SB1117 infection at 4 days post-infection (P ≤ 0.05 with fold change≥1.2 or ≤-1.2). Click here for file

Additional file 4 **Table S4. Target pathway of down-regulated genes in SL1344vs SB1117 infection group at 8 hours**. Listing target pathway of down-regulated genes in SL1344vs SB1117 infection group at 8 hours post-infection. Click here for file

Additional file 5 **Table S5. Target pathway of down-regulated genes in SL1344 vs SB1117 infection group at 4 days**. Listing target pathway of down-regulated genes in SL1344vs SB1117 infection group at 4 day post-infection. Click here for file
