# Supplementary material for: Eukaryotic signaling pathways targeted by Salmonella effector protein AvrA in intestinal infection in vivo
Source: BMC Microbiol. 2010 Dec 23;10:326. doi: 10.1186/1471-2180-10-326 (PMC3027599; doi:10.1186/1471-2180-10-326)
Supplement: Additional file 1 — Table S1. Primer sequence for qRT-PCR. Listing all primer sequences used in qRT-PCR (PDF file). PCR data were shown in Figure 3. [file 1471-2180-10-326-S1.PDF]

Table S1 Primer sequence for qRT-PCR

Gene name	Accession ID	Primer sequence	Product length (bp)
Junb	NM_008416	5'-TCACGACGACTCTTACGCAG-3'(F)	
		5'-CCTTGAGACCCCGATAGGGA-3'(R)	125
Muc1	NM_013605	5'-GGCATTCGGGCTCCTTTCTT-3'(F)	
		5'-TGGAGTGGTAGTCGATGCTAAG-3'(R)	132
Atf3	NM_007498	5'-GAGGATTTTGCTAACCTGACACC-3'(F)	
		5'-TTGACGGTAACTGACTCCAGC-3'(R)	110
Egr1	NM_007913	5'-TCGGCTCCTTTCCTCACTCA-3'(F)	
		5'-CTCATAGGGTTGTTCGCTCGG-3'(R)	227
SLC34a2	NM_011402	5'-CCTTGGCCCGAGTTGGAAAAT-3'(F)	
		5'-CTACAGGAGTCCCGTTGTCAT-3'(R)	130
MMP7	NM_010810	5'-CTGCCACTGTCCCAGGAAG-3'(F)	
		5'-GGGAGAGTTTTCCAGTCATGG3'(R)	175
Ube2c	NM_026785	5'-CTCCGCCTTCCCTGAGTCA-3'(F)	
		5'-GGTGCGTTGTAAGGGTAGCC-3'(R)	132
Rbm3	NM_016809	5'-CTTCGTAGGAGGGCTCAACTT-3'(F)	
		5'-CTCCCGGTCCTTGACAACAAC-3'(R)	103
SOCS3	U72673	5'-ATGGTCACCCACAGCAAGTTT-3'(F)	
		5'-TCCAGTAGAATCCGCTCTCCT-3'(R)	145
Caps14	NM_009809	5'-ACGCATGTTCCGTTACCTGAA-3'(F)	
		5'-CACCATGTGCCATGAGTACCA-3'(R)	152
beta-Actin	NM_007393	5'-TGTTACCAACTGGGACGACA-3'(F)	
		5'-CTGGGTCATCTTTTCACGGT-3'(R)	139
