# Supplementary material for: Eukaryotic signaling pathways targeted by Salmonella effector protein AvrA in intestinal infection in vivo
Source: BMC Microbiol. 2010 Dec 23;10:326. doi: 10.1186/1471-2180-10-326 (PMC3027599; doi:10.1186/1471-2180-10-326)
Supplement: Additional file 4 — Table S4. Target pathway of down-regulated genes in SL1344vs SB1117 infection group at 8 hours. Listing target pathway of down-regulated genes in SL1344vs SB1117 infection group at 8 hours post-infection. [file 1471-2180-10-326-S4.PDF]

Table S4

Target pathway of down-regulated Genes in SL1344 vs SB1117 infection groups at 8hr

Ingenuity Canonical Pathways	Downregulated	Molecules
PXR/RXR Activation	3/91 (3%)	CYP2C8,ALDH1A1, PRKAG2
PPARα/RXRα Activation	3/183 (2%)	CYP2C8, LPL, PRKAG2
CDK5 Signaling	2/93 (2%)	LAMB1, PRKAG2
FXR/RXR Activation	2/103 (2%)	OSTALPHA, FBP1
HIF1α Signaling	2/108 (2%)	SLC2A5, MMP7
LPS/IL-1 Mediated Inhibition of RXR Function	3/215 (1%)	CYP2C8,ALDH1A1,GSTA4
Type II Diabetes Mellitus Signaling	2/159 (1%)	SOCS2, PRKAG2
IL-9 Signaling	1/37 (3%)	SOCS2
AMPK Signaling	2/166 (1%)	PRKAG2, CHRNB4
Aryl Hydrocarbon Receptor Signaling	2/154 (1%)	ALDH1A1, GSTA4
mTOR Signaling	2/156 (1%)	RHOQ, PRKAG2
ATM Signaling	1/53 (2%)	FANCD2
Semaphorin Signaling in Neurons	1/52 (2%)	RHOQ
Role of BRCA1 in DNA Damage Response	1/61 (2%)	FANCD2
RAR Activation	2/181 (1%)	ALDH1A1, PRKAG2
CREB Signaling in Neurons	2/196 (1%)	GRIK5, PRKAG2
JAK/Stat Signaling	1/64 (2%)	SOCS2
NRF2-mediated Oxidative Stress Response	2/183 (1%)	GSTA4, EPHX1
Growth Hormone Signaling	1/70 (1%)	SOCS2
Hypoxia Signaling in the Cardiovascular System	1/70 (1%)	UBE2C
LXR/RXR Activation	1/86 (1%)	LPL
Melanocyte Development and Pigmentation Signaling	1/88 (1%)	PRKAG2
EIF2 Signaling	1/100 (1%)	EIF2A
IGF-1 Signaling	1/100 (1%)	PRKAG2
HMGB1 Signaling	1/98 (1%)	RHOQ
Type I Diabetes Mellitus Signaling	1/114 (1%)	SOCS2
Regulation of eIF4 and p70S6K Signaling	1/130 (1%)	EIF2A
