# Supplementary material for: Eukaryotic signaling pathways targeted by Salmonella effector protein AvrA in intestinal infection in vivo
Source: BMC Microbiol. 2010 Dec 23;10:326. doi: 10.1186/1471-2180-10-326 (PMC3027599; doi:10.1186/1471-2180-10-326)
Supplement: Additional file 5 — Table S5. Target pathway of down-regulated genes in SL1344 vs SB1117 infection group at 4 days. Listing target pathway of down-regulated genes in SL1344vs SB1117 infection group at 4 day post-infection. [file 1471-2180-10-326-S5.PDF]

Table S5

Target pathway of down-regulated Genes in SL1344 vs SB1117 infection groups at 4 days

Ingenuity Canonical Pathways	Downregulated	Molecules
RAN Signaling	2/23 (9%)	CSE1L, KPNA2
Eicosanoid Signaling	2/84 (2%)	PTGFR, PLA2G4C
TR/RXR Activation	2/97 (2%)	ADRB1, GH1
Lysine Degradation	3/234 (1%)	ECE2, ASTL, ADAMTS3
Aminophosphonate Metabolism	1/65 (2%)	PRMT5
Retinoic acid Mediated Apoptosis Signaling	1/44 (2%)	TNFRSF10A
Nucleotide Excision Repair Pathway	1/35 (3%)	GTF2H3
Calcium Signaling	3/206 (1%)	CHRNA4, RYR3, CASQ1
Selenoamino Acid Metabolism	1/77 (1%)	PRMT5
MIF Regulation of Innate Immunity	1/46 (2%)	PLA2G4C
Aminoacyl-tRNA Biosynthesis	1/83 (1%)	TARS2
Cysteine Metabolism	1/90 (1%)	CHST2
Death Receptor Signaling	1/64 (2%)	TNFRSF10A
β-alanine Metabolism	1/98 (1%)	UPB1
AMPK Signaling	2/166 (1%)	CHRNA4, ADRB1
Histidine Metabolism	1/120 (1%)	PRMT5
ERK5 Signaling	1/71 (1%)	CTF1
Growth Hormone Signaling	1/70 (1%)	GH1
Tyrosine Metabolism	1/202 (0%)	PRMT5
PPARα/RXRα Activation	2/183 (1%)	CHD5, GH1
cAMP-mediated Signaling	2/161 (1%)	ADORA3, ADRB1
Glycine, Serine and Threonine Metabolism	1/150 (1%)	TARS2
Ceramide Signaling	1/87 (1%)	ENPP7
